# Epidemiology of HIV Infection in Large Urban Areas in the United States

**DOI:** 10.1371/journal.pone.0012756

**Published:** 2010-09-15

**Authors:** H. Irene Hall, Lorena Espinoza, Nanette Benbow, Yunyin W. Hu, for the Urban Areas HIV Surveillance Workgroup

**Affiliations:** 1 Division of HIV/AIDS Prevention, National Center for HIV/AIDS, Viral Hepatitis, STD, and TB Prevention, Centers for Disease Control and Prevention, Atlanta, Georgia, United States of America; 2 STI/HIV Division, Chicago Department of Public Health, Chicago, Illinois, United States of America; 3 HIV Epidemiology Program, Los Angeles County Department of Public Health, Los Angeles, California, United States of America; Kenya Medical Research Institute, Kenya

## Abstract

**Background:**

While the U.S. HIV epidemic continues to be primarily concentrated in urban area, local epidemiologic profiles may differ and require different approaches in prevention and treatment efforts. We describe the epidemiology of HIV in large urban areas with the highest HIV burden.

**Methods/Principal Findings:**

We used data from national HIV surveillance for 12 metropolitan statistical areas (MSAs) to determine disparities in HIV diagnoses and prevalence and changes over time. Overall, 0.3% to 1% of the MSA populations were living with HIV at the end of 2007. In each MSA, prevalence was >1% among blacks; prevalence was >2% in Miami, New York, and Baltimore. Among Hispanics, prevalence was >1% in New York and Philadelphia. The relative percentage differences in 2007 HIV diagnosis rates, compared to whites, ranged from 239 (San Francisco) to 1239 (Baltimore) for blacks and from 15 (Miami) to 413 (Philadelphia) for Hispanics. The epidemic remains concentrated, with more than 50% of HIV diagnoses in 2007 attributed to male-to-male sexual contact in 7 of the 12 MSAs; heterosexual transmission surpassed or equaled male-to-male sexual transmission in Baltimore, Philadelphia, and Washington, DC. Yet in several MSAs, including Baltimore and Washington, DC, AIDS diagnoses increased among men-who-have sex with men in recent years.

**Conclusions/Significance:**

These data are useful to identify local drivers of the epidemic and to tailor public health efforts for treatment and prevention services for people living with HIV.

## Introduction

At the beginning of the human immunodeficiency virus (HIV) epidemic in the United States in the early 1980s, the majority of persons diagnosed with HIV were white and gay or bisexual men living in urban areas [1], [2]. While the epidemic continues to be primarily concentrated in urban areas—82% of reported acquired immune deficiency syndrome (AIDS) cases in 2006 were among persons who resided in metropolitan areas with population >500,000 [3]—overall the proportion of HIV infections attributed to male-to-male sexual contact has decreased (75% of AIDS diagnoses in 1983 compared with 47% in 2007) and racial/ethnic minorities comprise disproportionate fractions of persons affected by the disease [1], [4]. Such shifts in those impacted by the epidemic, in conjunction with increased prevalence due to wide availability of antiretroviral therapy, require shifts in prevention and care strategies. Similarly, local differences in the epidemiology of HIV require different approaches in prevention and treatment efforts.

Local HIV transmission dynamics may be influenced by differences in HIV prevalence among racial and ethnic groups or foreign-born populations at high risk for HIV infection, and behavioral factors conducive to HIV transmission. The proportion of minority populations differs between cities, which may affect HIV prevalence. Overall, the 2007 HIV diagnosis rate in 34 U.S. states among blacks/African Americans (76.7 per 100,000 population) was 8 times the rates among whites (9.2), and the lifetime risk of HIV diagnosis was estimated to be 1 in 16 for black/African American males and 1 in 30 for black/African American females compared to 1 in 104 for white males and 1 in 588 for white females [4], [5]. Among Hispanics, the HIV diagnosis rate was 3 times (27.7) that for whites, and the lifetime risk of HIV infection was estimated at 1 in 35 for Hispanic men and 1 in 114 for Hispanic females. Similarly, the drivers of the epidemic—male-to-male sexual contact, injection-drug use, and heterosexual contact—may differ between cities. While specific information on the size of each risk population is very limited, some estimates exist that show marked differences between urban areas. For example, the prevalence of injection-drug use has been shown to vary 12-fold across metropolitan areas overall, and by race/ethnicity groups and over time [6], [7]. It has been suggested that such differential impact of the HIV epidemic in geographic areas and at-risk populations puts HIV prevalence among these groups on par with some countries in sub-Saharan Africa [8].

We used data from national HIV surveillance to describe the epidemiology of HIV in the 12 metropolitan areas with the largest burden of HIV. These data are useful to identify local drivers of the epidemic and to tailor public health goals and planning for treatment and prevention services for people living with HIV.

## Methods

Since 1982, all 50 U.S. states and the District of Columbia report AIDS cases to the Centers for Disease Control and Prevention (CDC) in a uniform format. In 1994, CDC implemented data management for national surveillance of HIV integrated with AIDS case surveillance, at which time 25 states with confidential, name-based HIV surveillance started submitting case reports to CDC. Over time, additional states implemented name-based HIV surveillance and all states had implemented such surveillance by April 2008. All cases are reported to CDC without identifying information. Assessments of duplicate cases occur both on the state and national level (potential duplicates are identified based on soundex code [a phonetic algorithm for indexing names by sound, as pronounced in English] and selected demographic characteristics), and elimination of such cases occurs at the state level.

We used data on persons diagnosed with HIV infection (age >12 years) reported to CDC through June 2009 to describe the epidemiology of HIV in the 12 urban areas with the largest number of HIV diagnoses in 2007. Cases of HIV infection are counted by geographic area based on the person's residence at earliest known HIV diagnosis. The Metropolitan Statistical Areas (MSAs), as defined by the Office of Management and Budget [9]–[11], included were Atlanta-Sandy Springs-Marietta, GA; Baltimore-Towson, MD; Chicago-Naperville-Joliet, IL-IN-WI; Dallas-Fort Worth-Arlington, TX; Houston-Sugar Land-Baytown, TX; Los Angeles-Long Beach-Santa Ana, CA; Miami-Fort Lauderdale-Pompano Beach, FL; New York-Northern New Jersey-Long Island, NY-NJ-PA; Philadelphia-Camden-Wilmington, PA-NJ-DE-MD; San Francisco-Oakland-Fremont, CA; Tampa-St. Petersburg-Clearwater, FL; Washington-Arlington-Alexandria, DC-VA-MD-WV. For each of these MSAs, more than 1,000 HIV and/or more than 500 AIDS diagnoses were reported for 2007. We also describe the epidemiology of HIV for large cities/counties within these MSAs, including Atlanta, Baltimore (Baltimore City County), Chicago, Dallas, Fort Lauderdale, Houston, Los Angeles (Los Angeles County), Miami (Miami-Dade County), New York (Bronx, Kings, New York, Queens, and Richmond Counties), Philadelphia (Philadelphia County), San Francisco City and County (San Francisco County), Tampa, and Washington, DC.

We determined the distribution in HIV diagnoses (all diagnoses regardless of stage of disease at diagnosis) in the urban areas by race/ethnicity, age, sex, country of birth (U.S. vs. foreign born) and transmission category using information on persons diagnosed with HIV in 2007. This allowed for 18 months of follow-up time for reporting of diagnoses to CDC (cases reported through June 2009). Because several of the areas included in these analyses did not have name-based HIV reporting for the time required to calculate adjustment weights for reporting delays, analyses are not adjusted for reporting delays. Analyses by transmission category (male-to-male sexual contact [men who have sex with men, MSM]; injection drug use [IDU]; MSM and IDU; heterosexual contact with a person known to have, or to be at high risk for, HIV infection; and other) were adjusted for missing risk factor information [12], [13]. We also determined the number of persons living with HIV infection by race/ethnicity in the urban areas at the end of 2007.

Rates per 100,000 population were calculated for the MSAs overall and by race/ethnicity with population denominators based on official postcensal estimates from the U.S. Census Bureau [14]. Denominator data by race/ethnicity were available only for MSAs and counties; therefore, rates are not shown for cities that were not also defined by counties. Overall denominator data for cities not defined by counties were obtained from the U.S. Census Bureau estimates of the resident population for incorporated places over 100,000 and using the July 1, 2007 estimates [15]. Population denominators were not available to determine rates by transmission category.

It is well known that disparities in HIV burden exist among race/ethnicity groups. We explored inequities in HIV diagnosis rates within areas across populations using a relative measure of disparity recommended by the National Center for Health Statistics to compare variations in such inequities between areas [16]. We calculated the percentage difference in HIV diagnosis rates for each racial/ethnic group using the rates among whites as reference points ([rate of interest – rate among whites]/rate among whites*100) [16]. We also examined the correlation between MSA HIV prevalence and diagnosis rates by race/ethnicity and tested the significance of these correlations with the t-statistic.

To explore whether shifts in transmission dynamics have occurred over time, we determined trends in the proportion of persons diagnosed with AIDS by transmission category (percentage MSM and MSM-IDU) and race/ethnicity (percentage non-white) from 1985 through 2008. Analyses on AIDS diagnoses were adjusted for reporting delay and missing risk factor information [4], [12], [13].

## Results

In 2007, a total of 52,755 adolescents and adults were diagnosed with HIV in the United States and reported to CDC by the end of June 2009. Of these, 43,024 (81.6%) were living in urban areas with populations of 500,000 or more, and 25,997 (49.3%) were living in the 12 MSAs included in our analyses. The rates of diagnosis of HIV infection in the MSAs ranged from 22.8 per 100,000 population (Chicago MSA) to 77.2 (Miami MSA) (Table 1), and in cities/counties ranged from 29.2 (Los Angeles County) to 246.4 (Washington, DC). Forty-eight percent (Tampa MSA) to 85.7% (Baltimore MSA) of the new diagnoses were among non-whites. The rate of new diagnoses among blacks/African Americans ranged from 71.9 (Chicago MSA) to 197.8 (Miami MSA) in the MSAs and 79.3 (Los Angeles County) to 364.6 (Washington, DC) among cities for which rates were available. Hispanics comprised 2.4% to 42.1% of persons newly diagnosed with HIV in 2007, with a range of rates in the MSAs from 21.1 (Dallas) to 70.1 (Philadelphia). Hispanics had also high rates of HIV diagnosis in the MSAs of Baltimore (54.7), Miami (54.9), New York (53.2) and Tampa (48.6). While rates were not available for all the cities within the MSAs, in some cities the rates were higher for blacks/African Americans or Hispanic/Latinos than in the MSA as a whole.

**Table 1:** Numbers and rates (per 100,000 population) of adults and adolescents diagnosed with HIV infection in 2007, by race/ethnicity and area of residence, United States.

Area of residence	New Diagnoses of HIV Infection																		
Metropolitan Statistical Area City	American			Asian			Black/African American			Hispanic/Latino			White			Multiple Races/Other		Total	
	Indian/Alaska Native																		
	No.	%	Rate	No.	%	Rate	No.	%	Rate	No.	%	Rate	No.	%	Rate	No.	%	No.	Rate
Atlanta-Sandy Springs-Marietta, GA	5	0.3	49.5	9	0.5	5.3	1306	75.5	101.5	111	6.4	32.6	277	16.0	11.7	22	1.3	1,730	41.0
Atlanta				4	0.4		707	76.8		44	4.8		153	16.6		13	1.4	921	177.0
Baltimore-Towson, MD	3	0.2	49.6	9	0.6	11.0	1169	82.2	190.1	34	2.4	54.7	203	14.3	14.2	5	0.4	1,423	64.2
Baltimore				4	0.4	36.7	895	86.9	270.1	20	1.9	170.2	104	10.1	61.2	7	0.7	1,030	194.2
Chicago, IL-IN-WI	3	0.2	24.0	38	2.2	9.4	959	54.2	71.9	287	16.2	21.2	463	26.2	10.1	15	0.9	1,768	22.8
Chicago				23	1.8		753	57.5		211	16.1		305	23.3		18	1.4	1,310	46.2
Dallas, TX	4	0.3	17.3	19	1.5	8.4	569	43.9	84.5	250	19.3	21.1	446	34.4	16.4	4	0.3	1,297	26.6
Dallas				6	0.9		300	43.4		140	20.3		237	34.3		8	1.2	691	54.6
Houston-Baytown-Sugar Land, TX	3	0.2	23.3	15	1.1	5.9	731	53.8	99.2	333	24.5	24.5	275	20.2	13.5	3	0.2	1,360	30.7
Houston				12	1.1		590	54.5		280	25.9		196	18.1		4	0.4	1,082	50.0
Los Angeles, CA	7	0.3	23.8	128	4.7	8.6	590	21.9	78.0	1137	42.1	26.8	828	30.7	22.1	6	0.2	2,700	25.9
Los Angeles (Los Angeles County)				104	4.5	9.5	568	24.3	79.3	982	42.1	28.0	664	28.5	26.2	16	0.7	2,334	29.2
Miami, FL	3	0.1	39.1	12	0.3	12.9	1624	46.4	197.8	956	27.3	54.9	872	24.9	47.6	32	0.9	3,500	77.2
Miami (Miami-Dade County)				1	0.1	3.4	759	46.4	221.4	657	40.1	52.6	201	12.3	54.8	19	1.2	1637	81.9
Fort Lauderdale				3	0.5		286	44.6		73	11.4		270	42.1		9	1.4	641	351.1
New York, NY-NJ-PA	9	0.2	28.7	134	2.3	9.3	2783	47.9	106.9	1692	29.1	53.2	1095	18.8	13.1	95	1.6	5,815	36.9
New York				100	2.4	12.3	2004	48.3	123.2	1252	30.2	68.7	733	17.7	28.5	59	1.4	4,148	59.8
Philadelphia, PA-NJ-DE-MD	2	0.1	23.8	23	1.3	11.4	1053	60.2	112.1	193	11.0	70.1	461	26.3	13.7	16	0.9	1,750	36.2
Philadelphia				15	1.2	23.1	773	63.8	154.8	150	12.4	132.1	264	21.8	52.9	10	0.8	1,212	101.7
San Francisco, CA	2	0.2	17.1	66	6.1	8.5	290	26.8	95.5	219	20.2	33.7	481	44.5	28.1	17	1.6	1,082	30.4
San Fracisco City & County				41	7.1	18.5	79	13.7	174.8	117	20.3	124.3	323	56.0	97.4	17	2.9	577	81.4
Tampa-St. Petersburg-Clearwater, FL	1	0.1	13.9	4	0.4	7.1	286	31.1	123.3	145	15.8	48.6	478	52.0	28.5	5	0.5	920	40.1
Tampa				2	0.5		161	39.1		86	20.9		160	38.8		3	0.7	412	122.8
Washington, DC-VA-MD-WV	2	0.1	17.8	40	1.5	10.9	1954	73.7	174.1	213	8.0	45.4	423	16.0	18.1	16	0.6	2652	60.7
Washington DC				9	0.7	52.9	978	78.3	364.6	69	5.5	173.0	182	14.6	104.5	11	0.9	1,249	246.4

Because of the small populations of American Indian/Alaska Native populations in the cities, they are grouped with multiple races/other.

Because of small populations of Native Hawaiians and other Pacific Islanders they are grouped with multiple races/other.

At the end of 2007, a total of 793,348 adolescents and adults were diagnosed and living with HIV in the United States and reported to CDC by the end of June 2009. Of these, 400,814 (50.5%) were diagnosed in the 12 MSAs and included in our analyses. More than 1% of the population of the Miami MSA was living with HIV infection by the end of 2007 (1021.8 per 100,000 population) (Table 2). Overall HIV prevalence was also high in the MSAs of New York (806.3 per 100,000), Baltimore (777.6), DC (641.0) and San Francisco (622.7). In 9 of the 13 cities more than 1% of the population living with HIV infection, and in Atlanta, Baltimore, Fort Lauderdale, San Francisco, and Washington, DC the prevalence was more than 2%. In each MSA, more than 1% of the black/African American population was living with HIV at the end of 2007; prevalence was more than 2% in Miami, New York, and Baltimore. Among Hispanics, prevalence was above 1% in the MSAs of New York and Philadelphia. In Baltimore, Miami, San Francisco, and Washington, DC, the prevalence of HIV was higher among blacks/African Americans than in the respective populations of the MSAs, with prevalence the highest at 4.3% in San Francisco.

**Table 2:** Numbers and rates (per 100,000 population) of adults and adolescents living with HIV infection at the end of 2007, by race/ethnicity and area of residence, United States.

Area of residence	Persons Living with HIV																		
Metropolitan Statistical Area City	American			Asian			Black/African American			Hispanic/Latino			White			Multiple Races/Other		Total*	
	Indian/Alaska Native																		
	No.	%	Rate	No.	%	Rate	No.	%	Rate	No.	%	Rate	No.	%	Rate	No.	%	No.	Rate
Atlanta-Sandy Springs-Marietta, GA	27	0.1	267.2	68	0.3	39.9	13791	69.4	1,071.4	866	4.4	254.5	4969	25.0	209.1	147	0.7	19,871	470.2
Atlanta	17	0.2		28	0.3	.	7327	70.2		353	3.4		2631	25.2	.	73	0.7	10,431	2004.5
Baltimore-Towson, MD	28	0.2	462.9	47	0.3	57.3	13911	80.6	2,261.8	303	1.8	487.5	2837	16.5	198.4	125	0.7	17,251	777.6
Baltimore	21	0.2	1244.8	29	0.2	266.2	11153	86.1	3,365.5	165	1.3	1404.4	1506	11.6	886.4	74	0.6	12,948	2440.2
Chicago, IL-IN-WI	40	0.2	320.5	221	0.8	54.7	13420	51.2	1,005.9	4181	15.9	308.9	8063	30.8	176.2	295	1.1	26,222	338.6
Chicago	29	0.2		122	0.6	.	10728	53.8		3217	16.1		5580	28.0		267	1.3	19,945	704.1
Dallas, TX	41	0.2	177.2	171	0.9	75.7	6819	37.4	1,012.7	2965	16.3	250.4	8202	44.9	300.7	47	0.3	18,249	373.6
Dallas	19	0.2		78	0.7	.	4151	38.8		1795	16.8		4637	43.3		26	0.2	10,709	845.6
Houston-Baytown-Sugar Land, TX	25	0.1	193.8	172	0.9	67.9	9340	47.8	1,267.2	4017	20.6	295.3	5945	30.4	291.7	33	0.2	19,534	440.0
Houston	20	0.1		145	0.9	.	8217	50.3		3429	21.0		4481	27.5		26	0.2	16,323	738.9
Los Angeles, CA	121	0.3	412.0	1241	3.0	83.3	7801	18.7	1,031.8	16005	38.4	377.5	16216	38.9	432.1	243	0.6	41,650	399.3
Los Angeles (Los Angeles County)	105	0.3	483.5	1032	2.9	94.6	7395	20.9	1,032.6	13609	38.4	387.3	13020	36.8	513.7	223	0.6	35,400	442.7
Miami, FL	20	0.0	260.4	66	0.1	70.8	23737	51.3	2,891.3	11581	25.0	664.5	10253	22.1	559.3	650	1.4	46,307	1021.8
Miami (Miami-Dade County)	5	0.0	226.8	20	0.1	68.2	11391	48.1	3,323.3	8821	37.2	706.7	3163	13.3	862.4	304	1.3	23,704	1184.9
Fort Lauderdale	5	0.1		10	0.1		3509	48.1		715	9.8		2940	40.3		120	1.6	7,299	3998.0
New York, NY-NJ-PA	123	0.1	391.7	1266	1.0	88.2	60164	47.3	2,311.7	37811	29.8	1188.4	26284	20.7	314.4	1233	1.0	127,084	806.3
New York	87	0.1	486.2	1103	1.2	135.2	41796	45.8	2,570.0	29673	32.5	1627.2	17897	19.6	695.8	572	0.6	91,205	1315.4
Philadelphia, PA-NJ-DE-MD	33	0.1	392.9	132	0.5	65.5	15222	60.7	1,621.1	2815	11.2	1022.8	6641	26.5	196.8	237	0.9	25,098	518.4
Philadelphia	25	0.2	890.7	96	0.6	147.6	10841	65.7	2,171.6	1904	11.6	1676.2	3531	21.4	706.9	94	0.6	16,491	1384.2
San Francisco, CA	104	0.5	890.2	957	4.3	122.5	4900	22.1	1,613.4	3401	15.4	523.4	12618	57.0	737.9	173	0.8	22,155	622.7
San Francisco City & County	83	0.6	4487.6	630	4.4	283.7	1954	13.7	4,323.6	2141	15.0	2275.0	9339	65.5	2815.5	112	0.8	14,259	2005.5
Tampa-St. Petersburg-Clearwater, FL	16	0.2	221.5	32	0.3	56.5	3368	35.8	1,451.5	1217	13.0	407.6	4651	49.5	277.3	117	1.2	9,401	409.7
Tampa	7	0.2	.	6	0.1		2029	44.6		764	16.8	.	1694	37.2		49	1.1	4,549	1356.0
Washington, DC-VA-MD-WV	24	0.1	213.5	227	0.8	62.0	19813	70.8	1,765.4	1908	6.8	406.7	5799	20.7	248.3	197	0.7	27,992	641.0
Washington DC	12	0.1	873.8	41	0.3	240.9	10529	77.7	3,925.3	682	5.0	1709.7	2200	16.2	1263.2	84	0.6	13,556	2674.7

*Includes persons of unknown race/ethnicity.

Because of small populations of Native Hawaiians and other Pacific Islanders they are grouped with multiple races/other.

The relative percentage differences in 2007 HIV diagnosis rates in the MSAs, compared to whites, ranged from 239 (San Francisco) to 1239 (Baltimore) for blacks/African Americans; the percentage difference was less than 500% in San Francisco (217), Los Angeles (254), Miami (316), Tampa (333), Dallas (417), and more than 500% in Chicago (610), Houston (635), New York (716), Philadelphia (721), Atlanta (771), and Washington (861). For Hispanics/Latinos, the percentage difference ranged from 15 (Miami) to 413 (Philadelphia); the percentage difference was less than 100% in San Francisco (20), Los Angeles (22), Dallas (29), Tampa (70), Houston (82) and higher in Chicago (110), Washington (151), Atlanta (180), Baltimore (285), and New York (306). HIV diagnosis rates were lower for Asians than whites in all MSAs, and numbers were low in American Indian/Alaska Native populations and therefore, relative percentage differences are not presented. Diagnosis rates were correlated with HIV prevalence rates among blacks (r = .81, p<0.01), Hispanics (r = .76, p<0.01), and whites (r = .71, p = 0.01) but not among Asians or American Indians/Alaska Natives.

**Figure 1:**
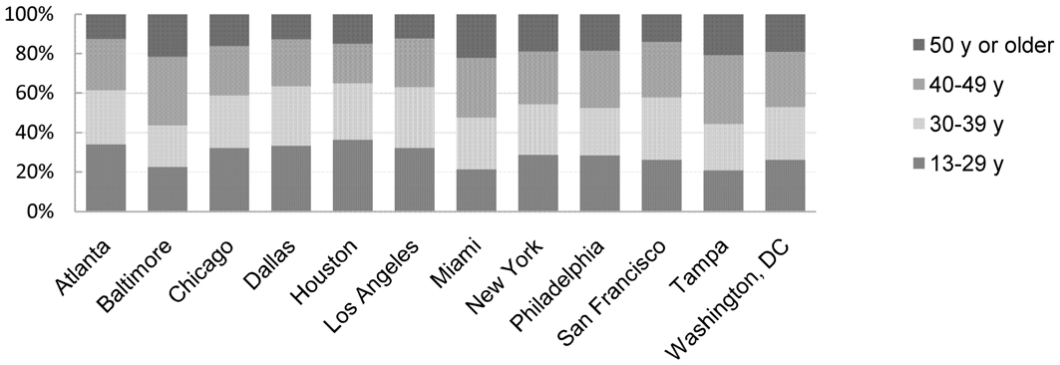
Percentage of adolescents and adults diagnosed with HIV, by area of residence and age, 12 U.S. Statistical Metropolitan Areas, 2007.

About a fifth of the persons diagnosed with HIV in Baltimore, Miami, and Tampa MSAs were aged less than 30 years at diagnosis, while more than 36% of diagnoses were among this age group in Atlanta, Chicago, Dallas, Houston, and Los Angeles (Figure 1). Conversely, in MSAs with the lowest percentage of diagnoses among the young more than 20% of diagnoses were among those aged 50 years or older. While information on country of birth was incomplete (data completeness ranged from less than 1% to almost 50%), some differences emerged with the largest percentage of persons diagnosed with HIV who were foreign-born in Los Angeles (21.1%), followed by Miami (14.9), San Francisco (10.3%), Houston (10.5%), New York (9.0%), Tampa (7.0%), and Chicago 5.8%) (data not shown).

About 14% (Los Angeles and San Francisco MSAs) to 36.5% (Baltimore MSA) of persons diagnosed with HIV in 2007 were women; the majority of these infections were attributed to heterosexual contact (Table 3). Baltimore MSA (30.3%) and San Francisco MSA (27.0%) had the highest percentages of women with reported IDU. Among men diagnosed with HIV, in the MSAs more than 70% were MSM except in Baltimore (52.4%), New York (66.8), Philadelphia (46.9%) and Washington (65.3%) (Table 4). Heterosexual contact accounted for about 20% of HIV infections among men in DC, Miami, and Baltimore MSAs, and 33.8% in Philadelphia. The distribution of HIV risk categories among men diagnosed with HIV in 2007 in the cities was similar to the distribution for the respective MSAs. Overall among all persons diagnosed with HIV, more than 50% of the HIV diagnoses in 2007 were attributed to male-to-male sexual contact in 7 of the 12 MSAs; heterosexual transmission surpassed or equaled male-to-male sexual transmission in Baltimore, Philadelphia, and Washington, DC.

**Table 3:** Numbers and percentages of adult and adolescent females diagnosed with HIV infection, by transmission category and area of residence, United States, 2007.

Metropolitan Statistical Area	IDU		Heterosexual contact		Other		Total
City	No.	%	No.	%	No.	%	No.
Atlanta-Sandy Springs-Marietta, GA	72	17.5	334	81.3	5	1.2	411
Atlanta	34	18.4	148	79.7	4	1.9	186
Baltimore-Towson, MD	158	30.3	360	69.2	3	0.5	520
Baltimore	126	32.5	261	67.2	1	0.4	389
Chicago, IL-IN-WI	90	24.7	270	73.9	5	1.5	365
Chicago	67	25.3	194	72.9	5	1.8	266
Dallas, TX	33	11.8	243	87.6	2	0.7	278
Dallas	13	8.8	131	90.9	0	0.3	144
Houston-Baytown-Sugar Land, TX	60	15.8	319	83.5	3	0.8	382
Houston	34	11.7	253	87.4	3	0.9	289
Los Angeles, CA	59	16.3	296	81.7	7	2.0	363
Los Angeles (Los Angeles County)	49	15.7	255	81.9	7	2.4	311
Miami, FL	100	10.1	887	89.7	3	0.3	989
Miami (Miami-Dade County)	38	9.0	386	90.9	1	0.1	425
Fort Lauderdale	21	12.3	146	87.5	0	0.2	167
New York, NY-NJ-PA	334	20.5	1276	78.4	18	1.1	1627
New York	227	20.5	872	78.7	9	0.9	1109
Philadelphia, PA-NJ-DE-MD	88	18.1	398	81.6	1	0.3	488
Philadelphia	57	16.6	285	83.4			342
San Francisco, CA	39	27.0	106	72.4	1	0.6	146
San Francisco City & County	21	41.8	28	57.6	0	0.6	49
Tampa-St. Petersburg-Clearwater, FL	39	17.4	185	82.3	1	0.3	225
Tampa	13	12.2	95	87.5	0	0.3	109
Washington, DC-VA-MD-WV	126	15.3	695	84.0	6	0.7	827
Washington, DC	72	21.4	265	78.3	1	0.3	338

Transmission category has been adjusted for missing risk factor information.

IDU, injection-drug use.

**Table 4:** Numbers and percentages of adult and adolescent males diagnosed with HIV infection, by transmission category and area of residence, United States, 2007.

Metropolitan Statistical Area	MSM		IDU		MSM/IDU		Heterosexual contact		Other		Total
City	No.	%	No.	%	No.	%	No.	%	No.	%	No.
Atlanta-Sandy Springs-Marietta, GA	1025	77.7	82	6.2	43	3.3	164	12.4	5	0.4	1,319
Atlanta	581	79.1	44	6.0	23	3.1	86	11.7	1	0.1	735
Baltimore-Towson, MD	473	52.4	221	24.4	32	3.5	175	19.4	3	0.3	903
Baltimore	312	48.8	180	28.1	23	3.5	124	19.3	2	0.3	641
Chicago, IL-IN-WI	1121	79.9	129	9.2	50	3.5	98	7.0	6	0.4	1,403
Chicago	826	79.1	104	10.0	39	3.7	73	7.0	3	0.3	1,044
Dallas, TX	872	85.6	46	4.5	28	2.7	71	6.9	3	0.3	1,019
Dallas	477	87.3	22	4.0	15	2.7	32	5.8	1	0.2	547
Houston-Baytown-Sugar Land, TX	700	71.6	70	7.2	38	3.9	169	17.3	1	0.1	978
Houston	584	73.7	38	4.8	30	3.8	140	17.7	1	0.1	793
Los Angeles, CA	2064	88.3	88	3.8	105	4.5	76	3.3	4	0.2	2,337
Los Angeles (Los Angeles County)	1794	88.7	64	3.2	90	4.4	72	3.6	3	0.1	2,023
Miami, FL	1755	69.9	127	5.1	87	3.4	538	21.4	4	0.2	2,511
Miami (Miami-Dade County)	826	68.2	68	5.6	42	3.5	274	22.6	2	0.1	1,212
Fort Lauderdale	354	74.7	24	5.0	22	4.6	73	15.4	1	0.3	474
New York, NY-NJ-PA	2796	66.8	664	15.9	120	2.9	603	14.4	5	0.1	4,188
New York	2071	68.1	453	14.9	90	3.0	421	13.8	4	0.1	3,039
Philadelphia, PA-NJ-DE-MD	591	46.9	204	16.2	39	3.1	427	33.8	0	0.0	1,261
Philadelphia	371	42.7	147	17.0	27	3.1	324	37.3	.	.	869
San Francisco, CA	728	77.8	61	6.5	85	9.1	62	6.6	1	0.1	936
San Francisco City & County	410	77.6	30	5.6	67	12.6	22	4.2	0	0.0	528
Tampa-St. Petersburg-Clearwater, FL	560	80.5	45	6.5	23	3.3	66	9.5	1	0.2	695
Tampa	236	77.9	20	6.6	9	3.1	38	12.4	0	0.0	303
Washington, DC-VA-MD-WV	1191	65.3	170	9.3	67	3.7	390	21.4	7	0.4	1825
Washington DC	590	64.7	104	11.4	39	4.3	176	19.3	2	0.3	911

Transmission category has been adjusted for missing risk factor information.

MSM, male-to-male sexual contact.

IDU, injection-drug use.

**Figure 2:**
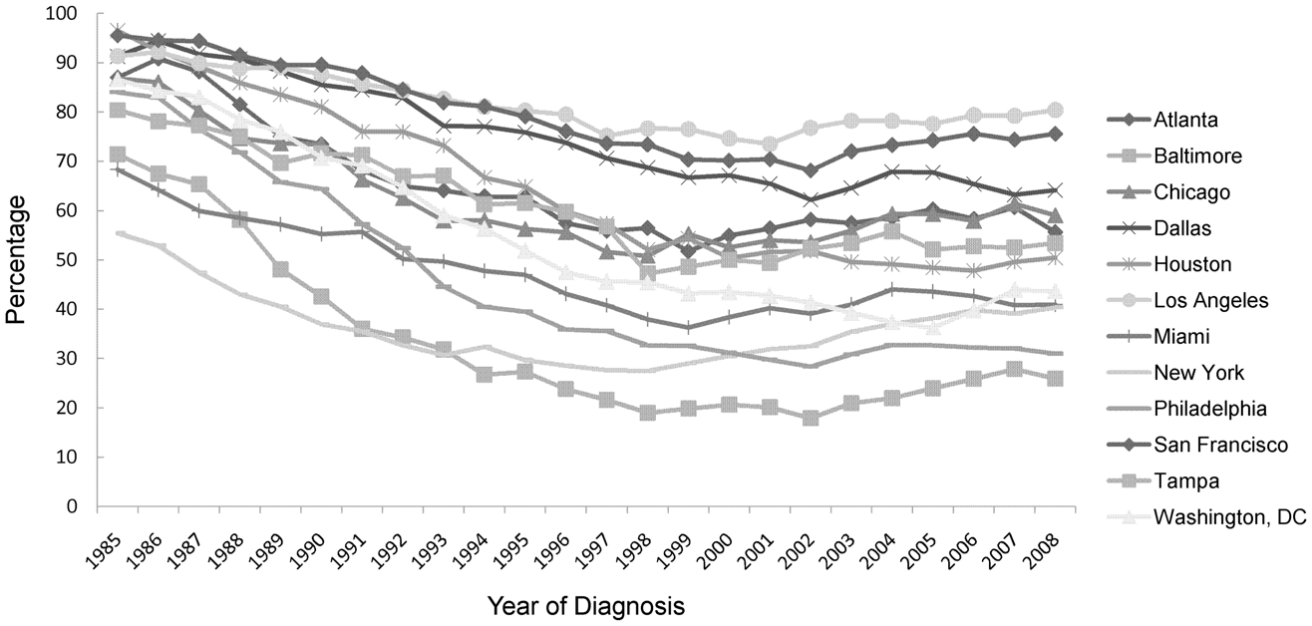
Percentage of AIDS cases attributed to men who have sex with men and to men who have sex with men and inject drugs, by area of residence and year of diagnosis, 12 U.S. Metropolitan Statistical Areas, 1985—2008.

**Figure 3:**
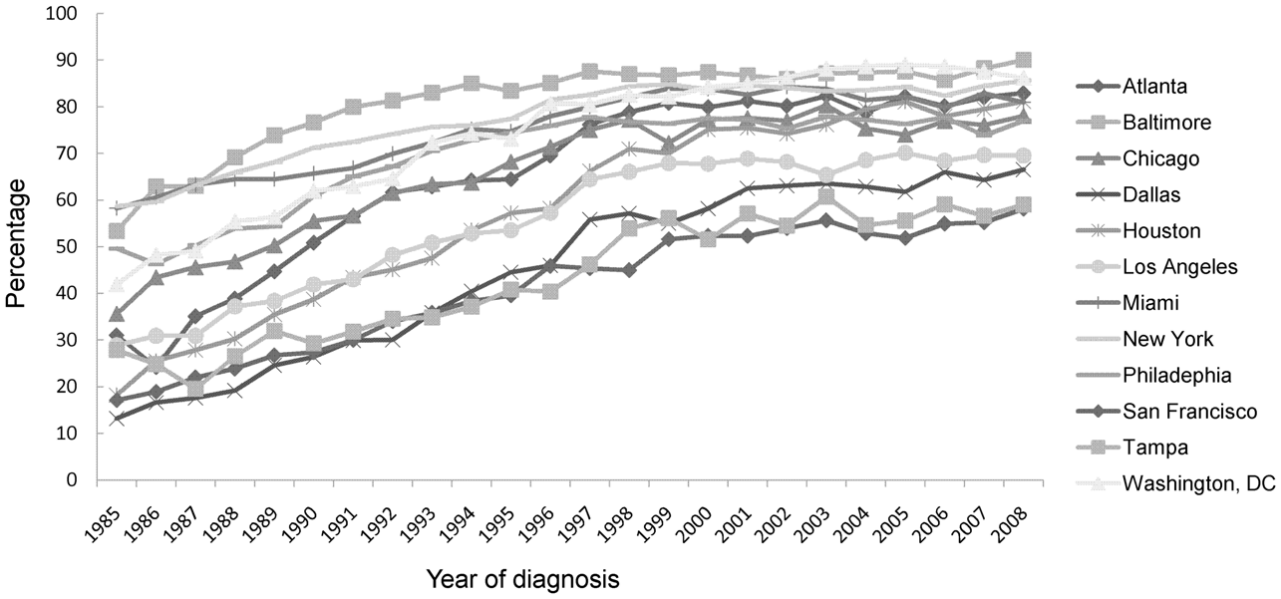
Percentage of AIDS cases among non-whites, by area of residence and year of diagnosis, 12 U.S. Metropolitan Statistical Areas, 1985—2008.

Over the course of the epidemic the composition of the population diagnosed with HIV and AIDS has changed. In all MSAs the percentage of AIDS cases attributed to MSM/MSM-IDU in the mid-1980s was 55% or more and the percentage decreased or leveled off in the late 1990s or early 2000s. However, the extent of the shifts in the local epidemics differed. The percentage of AIDS cases attributed to MSM/MSM-IDU decreased 12% in Los Angeles MSA, 20–30% in Dallas, New York and San Francisco, and more dramatically, by about 50% or more, in Washington, DC, Baltimore and Philadelphia (Figure 2). Increases in AIDS diagnoses in MSM/MSM-IDU were observed starting around 2002 in Los Angeles County, San Francisco, Chicago, Washington, DC, New York and Baltimore. Non-whites comprised an increasing percentage of persons diagnosed with AIDS in recent years, indicating racial/ethnic disparities in AIDS diagnoses persist and continue to grow (Figure 3).

## Discussion

This is the first report using national surveillance data to describe the epidemic of HIV in urban areas. In these 12 MSAs with a high burden of disease, more than 1% of the black population was living with HIV at the end of 2007 and prevalence was more than 2% in Miami, New York, and Baltimore. Among Hispanics, prevalence was above 1% in New York and Philadelphia. Prevalence generally was even higher in cities within MSAs, with HIV prevalence even among whites above 1% in Washington DC and above 2% in San Francisco. While racial/ethnic disparities exist in all areas, the relative percentage differences in 2007 HIV diagnosis rates varied widely. In addition, the drivers of the epidemic have shifted in some areas, with increased transmission now among heterosexual populations as well as MSM.

The World Health Organization categorizes the HIV epidemics of countries as low-level, concentrated, and generalized depending on HIV prevalence and diffusion of HIV transmission in different subpopulations [17], and some authors have suggested that some U.S. MSAs may be experiencing generalized epidemics [18]. In the past, with the majority of new HIV infections attributed to male-to-male sexual contact and the high HIV prevalence rates among MSM, findings indicated a concentrated HIV epidemic in the United States [19]–[21]. Overall, 53% of HIV diagnoses in 2007 were among MSM in 34 states with mature HIV reporting systems [4]. Our analyses show that the epidemic remains concentrated with more than 50% of the all HIV diagnoses in 2007 attributed to male-to-male sexual contact in 7 of the 12 MSAs. Heterosexual transmission surpassed or equaled male-to-male transmission in Baltimore, Philadelphia, and Washington, DC. However, increases in HIV transmission through heterosexual exposure may be fueled by men who have sex with men and women and IDU rather than indicate a generalized epidemic. In addition, our results reflect the trends in increasing incidence among MSM (19). In our analyses, we were not able to determine the HIV risk factors among sex partners of persons diagnosed with HIV.

HIV diagnosis and prevalence rates for the MSAs and the cities, where available, indicate marked differences between areas overall and among race/ethnicity subpopulations. Even areas that appear similar may be very different in terms of the drivers of the local epidemic. For example, while the HIV prevalence in the cities of Washington and San Francisco both exceeded 2%, and prevalence was high among blacks, Hispanics, and whites, the majority of HIV diagnoses were attributed to male-to-male sexual contact in San Francisco while in Washington the percentage of diagnoses attributed to male-to-male sexual contact and heterosexual contact was about the same.

There may be several explanations for differences in racial/ethnic disparities between areas. Lower disparity may be due to differences between areas in mixing between racial/ethnic populations and prevalence rates within racial/ethnic groups, the type of epidemic (e.g., San Francisco and Los Angeles continue to have concentrated epidemics with the majority of diagnoses attributed to male-to-male sexual contact), or better penetration of HIV testing among all race/ethnicity groups with linkage to care and fewer undiagnosed persons. For example, the HIV prevalence rate among whites is relatively high in Miami and San Francisco and may explain why these areas have relatively lower disparities. In some areas a higher proportion of persons diagnosed with HIV was born outside of the United States. However, it is unclear if they were infected in the United States or abroad. In general, foreign-born persons are less likely to have health insurance, and may be more vulnerable to HIV infection where male dominant relationship dynamics exist, men are targeted by sex workers, or behaviors change as it is easier to engage with multiple sex partners in the new country [22]. Women, on the other hand, may have more access to health and social services due to reproductive services.

Correlations between HIV prevalence and diagnosis rates, in our analysis observed for blacks, Hispanics, and whites, are expected as persons would be more likely to encounter HIV-positive partners in areas with higher prevalence. However, a goal to reduce prevalence is unlikely met in the near future, as prevalence is expected to rise as people with HIV live longer with better antiretroviral treatments regimens and with earlier initiation of treatment [23]. Therefore, the nearer goal should be to assure the early detection of HIV infection and diagnosis of infection among persons unaware of their infections status, and linkage to care and prevention services to reduce transmission rates [24].

There is evidence that persons aware of their HIV-positive status reduce risk behaviors and can therefore impact transmission rates [25]. However, about 21% of persons infected with HIV are unaware of their infection [26] and not all who need treatment are receiving it; these persons contribute disproportionately to HIV transmission rates through risk behavior and high viral loads. To identify all HIV infections among the undiagnosed and as early as possible, CDC recommends routine HIV screening in all health-care settings for persons aged 13—64 years and pregnant women and retesting at least annually for all persons at high risk for HIV [27]. CDC has expanded the HIV testing initiative to increase testing and knowledge of HIV status and to reach more U.S. jurisdictions and populations at risk, including African-American men and women, gay and bisexual men, and male and female Latinos and injection-drug users [28]. Many cities have also implemented intensified testing and prevention efforts coupled with public education campaigns. For example, the New York City Department of Health and Mental Hygiene is implementing a large-scale initiative, The Bronx Knows, to increase voluntary HIV testing and provide access to quality care and prevention [29]. The District of Columbia has implemented intensified testing, linkage to care, free condom distribution, and needle exchange to address the high HIV transmission in the District [30].

In addition, proven behavioral interventions for high-risk populations exist [31] and such interventions have shown to reduce risk behavior by 20 to over 40% [32]. Therefore, interventions should also include education campaigns and interventions for HIV-negative persons at risk for infection. However, while many of these interventions have been implemented in prevention programs across the country, evidence suggests individual interventions reach only a low proportion of MSM [33].

Our analyses are subject to several limitations. Because we were not able to adjust for reporting delays, we may have underestimated the number of new HIV diagnoses in 2007 and the number of persons living with HIV; the latter may also be an underestimate in areas that have recently transitioned from code to name-based HIV reporting and that have been unable to re-ascertain all persons with HIV with names (the code-based data are not reported to CDC). Our analyses also do not include persons who have not been diagnosed. Information on country of birth was incomplete in some areas, ranging up to 49% of cases missing this information. Finally, we were not able to calculate rates for all cities as denominator data were not available by race/ethnicity for all of them.

In summary, we found that epidemic profiles differ in local areas of the United States. These data are useful to identify local drivers of the epidemic and to tailor public health efforts for treatment and prevention services for people living with HIV. HIV prevention efforts should include, as appropriate for the local population, HIV testing and prevention interventions with HIV-positive persons and persons at high risk for infection.
